# Designing a children’s health exposomics study protocol: The CHILDREN_FIRST multi-country prospective cohort using multi-omics and personalized prevention approaches

**DOI:** 10.1371/journal.pone.0326641

**Published:** 2026-04-27

**Authors:** Corina Konstantinou, Georgia Soursou, Samuel Abimbola, Pantelis Charisiadis, Angelos Kyriacou, Theofano Modestou, Michalis Tornaritis, Charalambos Hadjigeorgiou, Agapios Agapiou, Efstathios A. Elia, George Milis, Alexis Kyriacou, Lygia Eleftheriou, Zoi Tsimtsiou, Pantelis Natsiavas, Or Duek, Idan Menashe, Nathalia Bilenko, Itamar Grotto, Enkeleint A. Mechili, Mònica Guxens, Costas A. Christophi, Constantinos Deltas, Konstantinos C. Makris

**Affiliations:** 1 Cyprus International Institute for Environmental and Public Health, School of Health Sciences, Cyprus University of Technology, Limassol, Cyprus; 2 biobank.cy, Centre of Excellence in Biobanking and Biomedical Research, University of Cyprus, Nicosia, Cyprus; 3 Research and Education Institute of Child Health, Nicosia, Cyprus; 4 Department of Chemistry, University of Cyprus, Nicosia, Cyprus; 5 PHOEBE Research and Innovation LTD, Nicosia, Cyprus; 6 CP. Food Lab, Nicosia, Cyprus; 7 Department of Hygiene, Social-Preventive Medicine and Medical Statistics, School of Medicine, Aristotle University of Thessaloniki, Thessaloniki, Greece; 8 Institute of Applied Biosciences, Centre for Research and Technology Hellas, Thessaloniki, Greece; 9 Ben-Gurion University of the Negev, Beersheba, Israel; 10 Department of Healthcare, Faculty of Health Sciences, University of Vlora, Vlora, Albania; 11 ISGlobal, Barcelona, Spain; 12 ICREA, Barcelona, Spain; 13 Universitat Pompeu Fabra, Barcelona, Spain; 14 Spanish Consortium for Research on Epidemiology and Public Health (CIBERESP), Instituto de Salud Carlos III, Madrid, Spain; 15 Department of Child and Adolescent Psychiatry/Psychology, Erasmus MC, University Medical Centre, Rotterdam, the Netherlands; 16 Department of Rehabilitation Sciences, School of Health Sciences, Cyprus University of Technology, Limassol, Cyprus; 17 School of Medicine, University of Cyprus, Nicosia, Cyprus; Federal University of Santa Maria: Universidade Federal de Santa Maria, BRAZIL

## Abstract

Non-communicable diseases (NCDs) account for ~71% of all deaths globally, including 15 million premature deaths each year (deaths between 30–69 years of age). Instead of waiting until disease manifestation, focusing on the origins of NCDs during childhood offers a critical window of disease prevention and control. The CHILDREN_FIRST international cohort observatory study aims to investigate how the spatio-temporal evolution of the children’s exposome profiles in the Mediterranean region influences early-life programming of chronic disease risk during the critical window of susceptibility in primary school years (6–11 years of age). The study protocol adopts the human exposome framework integrated with a personalized prevention approach, using multi-omics platforms and advanced machine learning algorithms implemented across Mediterranean countries, namely Cyprus, Greece, and Albania. The cohort will consist of children enrolled in the first grade of primary school, who will undergo annual follow-up assessments until completion of primary education. During the annual assessments, children’s exposome parameters from the three main exposome domains will be evaluated using different assessment types, i.e., molecular biomarkers of exposure/effect, sensors, and questionnaires. Standardized biospecimen and data collection methods will be employed following harmonized standardized operating procedures. The reference model of Observational Medical Outcomes Partnership – Common Data Model (OMOP-CDM) developed and maintained as part of the Observational Health Data Sciences and Informatics (OHDSI) initiative will be used to conduct federated data analysis. This CHILDREN_FIRST study protocol is a human exposome-based initiative to establish a long-term prospective cohort infrastructure for biomedical research on children’s health in the Mediterranean region. The cohort’s exposome-based findings will systematically feed into the evaluation and design of chronic disease prevention programs. Expected results would inform evidence-based policy making and the development of health interventions for reducing the risk of NCDs in childhood and later in adult life.

## 1 Introduction

Non-communicable diseases (NCDs) account for ~71% of all deaths globally, including 15 million premature deaths each year (deaths between 30–69 years of age), thus, undermining workforce productivity and impeding economic growth [1]. Among adolescents aged 10–24 years, NCDs are responsible for 86.4% of all years lived with disability (YLDs) and 38.8% of total deaths [2]. Considering the global cancer burden, the leading risk factors with the highest number of age-standardized disability-adjusted life years (DALYs) remained the same between 2010 and 2019, with smoking, alcohol use and high body mass index (BMI) identified as the top contributors [3].

Global efforts adopt a life course approach to NCD prevention, acknowledging the value of addressing risk factors early in life [4–6]. Instead of waiting until the manifestation of disease’s symptoms, the focus on the origins of NCDs during childhood, offers a critical window of disease prevention and control for effective preventive interventions, rather than relying solely on prevention or treatment strategies later in adulthood. Children exhibit increased susceptibility to environmental exposures compared to adults because of their rapid development, differences in behaviors and metabolism, and indirect/passive exposures to multiple environmental stressors via their parents’ habits and lifestyle [7].

On the fetal programming of chronic disease, several birth-pregnancy cohorts have mostly collected baseline exposure data during the prenatal period and followed up during early life, while other cohorts extended their follow-ups into the primary school years and beyond. In particular, the age span of 6–11 years, being the first years of school life, carries some unique features. In effect, children enter primary school, spending more time in organized educational settings (schools), compared to when they were younger, and begin different hobbies, social interactions and friendships, while their learning experience gets steeper and often versatile in content and types [8]. Based on the predicted obesity prevalence rates among 2-year-old children from the CHOICES simulation model, a steeper increase in obesity prevalence was estimated for ages between 5–11 years old [9] than younger age groups or older than 12 years of age. Moreover, longitudinal studies using data from seven-year-old children (n = 2438) have shown that three out of six identified lung function risk trajectories – that contribute to 75% of chronic obstructive pulmonary disease (COPD) in adulthood – originate in childhood [10].

Holistic methodological frameworks, such as the human exposome concept and its exposomic tools are essential for supporting the implementation of children’s health policies [11]; such frameworks would allow us to better understand the complex associations between environmental exposures and trajectories of healthy growth and development in children. The human exposome encompasses all environmental exposures from conception onwards, with the associated biological response [12,13]. It was proposed in an attempt to elucidate the role of the environment in the development of chronic diseases [14] given that environmental factors may contribute up to 80–90% of NCD risks [15]. In conjunction with the human exposome concept and its utility, the precision prevention approach within the personalized medicine paradigm emerges as a new medical model that seeks to characterize individual phenotypes and genotypes to determine disease predisposition and to deliver timely prevention [16]. When applied to children’s populations, precision prevention would study differences in children’s genetic makeup, environmental exposures, and lifestyle factors. This approach not only provides knowledge about enhanced risk profiling and population stratification but also holds promise for informing more effective individualized interventions to improve child health outcomes [17].

There are several children’s exposome projects to date, with most of them enrolling mothers during pregnancy [18,19]. Such cohort exposome studies collect repeated in time sample/data prenatally, but they often go by a single assessment point during the 6–11 age period. Some children’s exposome studies pool data from existing birth cohorts, limiting the uniformity of exposure and outcome measurements and they may typically vary in recruitment and follow-up timings, children’s age at the time of the examination, data completeness, and biospecimen measurement types and tools. Harmonization of data collection tools and procedures across diverse settings and cohorts that pool data from heterogeneous cohorts and their populations is a daunting task [20,21].

**Fig 1:**
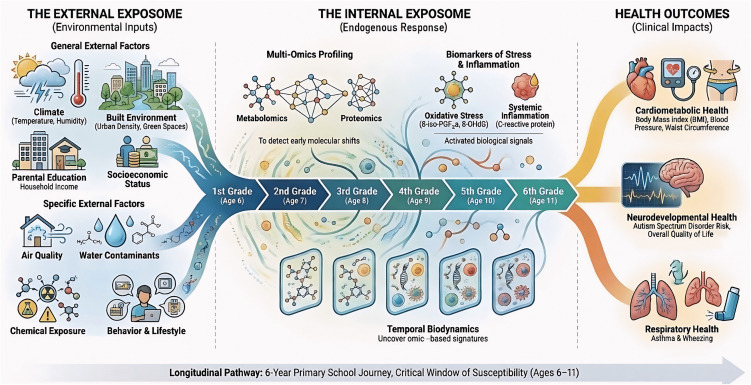
CHILDREN_FIRST conceptual framework. Adapted from a figure generated with NotebookLM [22].

In response to these methodological challenges in children’s health, we designed a multi-country site exposomic longitudinal cohort children’s study, during a critical window of vulnerability, using *a priori* harmonized protocols, that would enable its future adaptation or replication to a different population and setting around the globe (Fig 1). As a case study, we present the CHILDREN_FIRST exposome-based study protocol for the multi-country prospective cohort study focused on children in Mediterranean populations. This longitudinal study aims to investigate how the spatio-temporal evolution of the children’s exposome profiles in the Mediterranean region influences the early-life programming of chronic disease risk during the critical window of susceptibility present in the primary school years (6–11 years of age). The CHILDREN_FIRST study adopts the human exposome framework and its exposomic tools together with a personalized prevention approach, using multi-omics platforms and advanced machine learning algorithms.

The specific objectives of the CHILDREN_FIRST exposome-based prospective cohort study are to: **i)** characterize the spatio-temporal variability of the targeted/untargeted multi-exposomic profiles of primary school children in Cyprus, Greece, and Albania, using standardized sample/data collection, biomarker and multi-omics capabilities and machine learning data processing algorithms, **ii)** evaluate the effect of single and multiple environmental exposures on early-life biological programming linked to chronic disease risk, by applying validated biomarkers of exposure and effect, related to cardio-metabolic, neurodevelopmental, and respiratory outcomes and their early precursors, such as inflammation and oxidative stress/damage, including multi-omics, meet in the middle capabilities, and **iii)** generate NCD risk prediction groups of children using precision prevention approaches that include the spatio-temporal profiling of both genetic and non-genetic stressors, such as environmental, social, behavioral, metabolic, and psychosocial risk factors and multi-omics platforms.

## 2 Materials and methods

### 2.1 Study design

**Fig 2:**
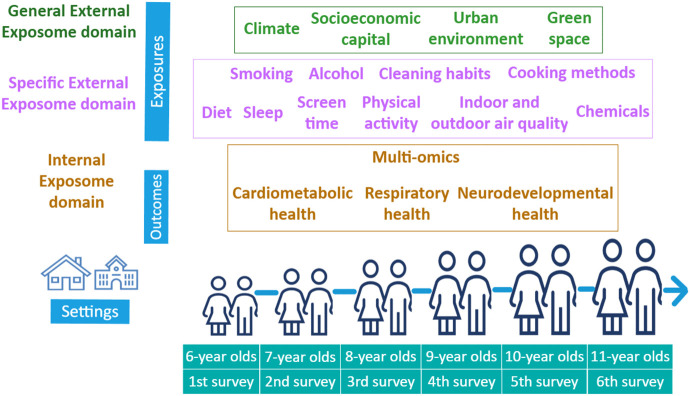
CHILDREN_FIRST study design. Annual children’s exposome assessments during primary school years (first grade to sixth grade).

CHILDREN_FIRST is a multi-site, longitudinal cohort study to be implemented across Cyprus, Greece and Albania with the prospect to include other Mediterranean sites in the future. Schools will be the main setting for recruitment, assessment and reporting back, by enrolling children in the first grade of primary school, who will undergo annual follow-up assessments until completion of primary education (Fig 2). During the annual assessments, exposome parameters from the three main exposome domains will be evaluated.

The study protocol has been developed in accordance with the SPIROS checklist [23]. Key methodological elements addressed, include the study design and setting, participant eligibility and recruitment procedures, standardized assessment of exposures and outcomes and harmonized data collection across the three country sites. Strategies to address potential sources of bias, including confounding and loss to follow-up, as well as statistical analysis and data management procedures, are also described. A completed SPIROS checklist is provided in Supplementary File 2 (S2 File) for transparency and completeness.

### 2.2 Study population

Randomly selected public primary schools will be the study’s main recruitment setting. All first-grade students attending the randomly selected schools and residing in the country for at least the past year will be eligible for inclusion. The number of participants recruited from each area will be proportionate to the population distribution of children, taking also into account the degree of urbanization and the administrative units of each country (e.g., districts, municipalities, etc.) (more about recruitment in S1 File).

### 2.3 Study procedures

Annual assessments of the children’s exposome will be conducted from the first through the sixth grade of primary school. Standardized human sample and data collection methods will be employed in all countries following standardized operating procedures (more about Assessment methods and Questionnaires list in S1 File). Table 1 outlines the variables to be assessed annually beginning at baseline (aggregated to a broad class variable), the corresponding assessment methods (e.g., electronic questionnaires, biospecimen) and their classification within the exposome framework (e.g., general external, specific external, or internal exposome domain).

**Table 1:** Overview of variables, assessment methods and exposome domains to be measured in the CHILDREN_FIRST prospective cohort study.

Variable group	Assessment method	Exposome domain*
Climate (ambient air temperature and relative humidity)	Sensors in schools	General external
Built environment (e.g., population density, building density, street connectivity)	GIS linkage to local spatial data, secondary data	General external
Neighborhood quality of life (e.g., health care access, life in the neighborhood and green urban spaces)	Questionnaire	General external
Socioeconomic capital (e.g., parental education and employment, household composition)	Questionnaire	General external
Outdoor air quality (e.g., CO_2_, PM, VOCs)	Sensors and passive samplers in schools	Specific external
Indoor air quality (e.g., CO2, PM, temperature, humidity, noise, UV, VOC)	Sensors and passive samplers in schools, questionnaire on school/classroom/home characteristics	Specific external
Residential and school environment (e.g., noise, traffic, ventilation, building materials, heating sources, cleaning practices, cooking methods, ALAN)	Questionnaire on school/classroom/home characteristics, satellite-based images of residences for outdoor ALAN	Specific external
Water contaminants (e.g. THMs, PFAS)	Tap water samples from houses and schools	Specific external
Child lifestyle, e.g., physical activity, sedentary behavior, screen time, diet, sleep patterns, chronotype	Questionnaire	Specific external
Parental lifestyle (e.g., smoking, alcohol use, occupation)	Questionnaire	Specific external
Biomarkers of exposure (e.g., pesticides, metals, bisphenols, VOCs)	Urine and saliva samples^^^	Specific external
Background characteristics (e.g., age, sex)	Questionnaire	Internal
Systems biology (e.g., omics)	Urine and saliva samples	Internal
Oxidative stress/damage and inflammation	Urine samples	Internal
Micronutrients	Urine samples	Internal
Circadian function (e.g., corticosteroids)	Urine and saliva samples	Internal
Liver and renal function	Urine samples	Internal
Medical history (e.g., health status, medicine use pregnancy/breastfeeding practices)	Questionnaire	Internal
Intrinsic properties (e.g., weight, height, waist circumference, birth weight)	Anthropometric measurements, questionnaire	Internal
Psychosocial health (e.g., stress, allostatic load, etc.)	Questionnaire	Internal
Neurodevelopmental disorders (e.g., ASD)	Questionnaire	Internal
Cardio-metabolic outcomes (e.g., BMI, blood pressure)	Anthropometrics, Sphygmomanometer	Internal
Respiratory health (e.g., asthma, wheezing)	Questionnaire	Internal

* Exposome domains based on [13].

^ Non-invasive biospecimen matrices are preferred instead of blood, because of the more frequent repeated measures nature of the CHILDREN_FIRST protocol.

Abbreviations: GIS: Geographic Information System, CO_2_: Carbon dioxide, PM: Particulate matter, VOCs: Volatile organic compounds, ALAN: Artificial light at night, THMs: Trihalomethanes, PFAS: Per- and polyfluoroalkyl substances, ASD: Autism spectrum disorder, BMI: Body mass index.

In case of budgetary or logistical constraints, the exposures and analyses will be prioritized based on predefined criteria, including assessment of cost, variability within the children’s population, scientific importance, and policy relevance. Priority will be given to low-cost exposures.

While other biospecimens, such as hair or nails, are not excluded, the main biospecimens that will be collected in annual assessments will be urine and saliva samples. Hair and nail samples may be collected in follow-up assessments, following development of appropriate SOPs that will be agreed upon by all country sites. The biological samples will be stored in the Biobank of the University of Cyprus, which is part of the biobank.cy Center of Excellence in Biobanking and Biomedical Research, and in respective biobanks of participating countries to enable future analyses. Samples analyses will follow a similar predefined hierarchy, so in case of budgetary constraints, high-cost laboratory analyses, such as omics analyses, will be postponed until funding is secured.

### 2.4 General external domain

Climatic parameters, specifically air temperature and relative humidity, will be monitored using sensors installed in outdoor areas of the participating schools. Built environment characteristics, including population density, building density, and street connectivity, will be assessed through Geographic Information System (GIS) linkage to local spatial datasets and relevant secondary data sources. Standardized questions based on validated questionnaires will be used for the assessment of demographic characteristics and neighbourhood quality of life (e.g., life in the neighbourhood and green urban spaces) [24], health care access [25], sociodemographic variables (e.g., social position of children using multiple sociodemographic variabls from EU SILC, such as parents’ employment and education status, parental age, country of birth (grandparents, parents, and child), language spoken at home, years living in area, household income, number of people in household, family/cohabitation status, address, and contact info) [24,26,27].

### 2.5 Specific external domain

#### 2.5.1 Air quality.

Air quality parameters will be monitored in classrooms of participating schools (and potentially also in children’s residences) for a minimum of one school week, each year. Measurements will include PM_10_, PM_2.5_, air temperature, relative humidity, CO₂, as well as volatile organic compounds (VOCs), such as benzene, toluene, ethylbenzene, xylene (BTEX), and chloroform.

#### 2.5.2 Water pollutants.

All four trihalomethanes (THMs) species - chloroform (TCM), bromodichloromethane (BDCM), dibromochloromethane (DBCM), and bromoform (TBM) – will be measured in the collected tap water samples from the participating houses and schools. The THMs analysis will be conducted according to a previously published method [28]. Per- and polyfluoroalkyl substances (PFAS), halogen oxyanions, as well as, microbiological parameters will also be assessed in tap water.

#### 2.5.3 Chemical contaminants.

Numerous chemicals could be targeted here. Indicatively, we plan on using urinary biomarkers of exposure to pesticides, including two pesticide metabolites: 3-phenoxybenzoic acid (3-PBA), a biomarker of pyrethroid exposure, and 6-chloronicotinic acid (6-CN), a metabolite of neonicotinoid pesticides using a gas-chromatographic-tandem mass spectrometric (GC–MS/MS) method based upon modifications of two existing protocols [29,30]. Urinary concentrations of lead and cadmium will also be measured using the Centers for Disease Control and Prevention (CDC) multi-element ICP-DRC-MS method No. 3018.3 [31]. Urine samples will be analyzed for free and total forms of bisphenols, such as bisphenol A (BPA), bisphenol F (BPF) or bisphenol S (BPS) and three chlorinated BPA derivatives: 3-chlorobisphenol A (ClBPA), 3,5-dichlorobisphenol A (3,5-Cl₂BPA), and 3,3’-dichlorobisphenol A (3,3’-Cl₂BPA), using a modified version of a previously published urinary BPA analysis protocol [32]. A suite of VOCs will be assessed in saliva samples.

#### 2.5.4 School and home environment.

Standardized questions based on validated questionnaires will be used for the assessment of residential environment and home exposures [24,27,33] and school building/classroom characteristics that potentially have an impact on indoor air quality [34]. Indoor artificial light at night (ALAN) will be assessed using a subjective measure defined as the level of light in the bedroom during sleeping time [35]. The response will be a four-digit Likert scale: a) total darkness, b) almost dark, c) dim light, and d) quite illuminated. For the evaluation of outdoor ALAN, calibrated satellite-based images of children’s residences with high-quality spatial resolution during the study period will be used.

#### 2.5.5 Parental and child lifestyle.

Standardized questions based on validated questionnaires will be used for the assessment of parents’ lifestyle (e.g., occupation, smoking and alcohol habits, household cleaning activities, and cooking methods at home) [27] and child’s lifestyle (e.g., physical activity, screen time, diet, cosmetic and hygiene products use, activities at home/school, and drinking water habits) [27,36–40], chronotype [41], smoking and alcohol habits (relevant for children aged 10–11) [27], and exposure directly prior (past 24 or 48 hours) to the sampling [27].

### 2.6 Internal exposome domain

#### 2.6.1 Multi-omics.

Urine and saliva samples will be invaluable for multi-omics profiling to explore molecular mechanisms, linking exposures with outcomes and capturing both intermediate biological responses and early biological effect biomarkers, across the cohort. Urine samples will be subjected to untargeted GC–MS metabolomics analysis [42]. Metabolomics will be used as an intermediate biological layer between biomarkers of exposure and biomarkers of effect. Saliva samples will be used for transcriptomic profiling to detect RNA biomarkers associated with early biological programming and disease susceptibility [43]. Extracting RNA from saliva can offer insight into inflammation pathways, which are usually modified before signs of disease appear. Urine proteomics will be used to identify protein biomarkers related to metabolic and systemic functions [44]. Proteomic signatures can reflect early-stage biomarkers, thus identifying early deviations from a healthy status.

#### 2.6.2 Oxidative stress/damage and inflammation.

Competitive ELISA or mass spectrometry will be used to determine urinary concentrations of oxidative stress/damage and inflammation biomarkers (i.e., 8-iso-PGF2α, 8-OHdG, C-reactive protein, 4-HNE). Creatinine-corrected biomarker values will be calculated after measurements of urinary creatinine using the colorimetric Jaffé method [45].

#### 2.6.3 Micronutrients.

Urine samples will be analysed for total iodine following the CDC Environmental Health protocol ITU004A [46] using inductively coupled plasma mass spectrometer (ICP-MS; Thermo X Series II, Thermo Scientific). Based on the WHO classification scheme [47], for school-age children (≥6 years of age), an adequate iodine level is defined as a population median urinary iodine concentration of 100–199 μg/L, whereas a population median of <100 μg/L indicates that the population’s iodine intake is insufficient. Intake of other micronutrients, including vitamins, iron and selenium will be estimated based on dietary recall data.

#### 2.6.4 Corticosteroids and circadian rhythm markers.

GC-MS methods will be used to determine urinary/salivary concentrations of corticosteroids, including, cortisol, estradiol, testosterone, [48] as well as melatonin as a marker of circadian rhythm.

#### 2.6.5 Liver and renal function.

Biomarkers of liver and renal function that will be measured in urine samples using conventional biochemical spectrophotometry assay, include glucose, bilirubin, ketones, specific gravity, blood, pH, protein, urobilinogen, nitrite, creatinine, leucocytes, albumin, total protein and calcium with conventional biochemical spectrophotometry assay.

#### 2.6.6 Intrinsic properties and medical history.

Standardized questions based on validated questionnaires will be used for the assessment of each child’s intrinsic characteristics (e.g., age, sex) [27] and medical history (e.g., parents’ diseases, child’s diseases, medicine use, birth anthropometrics and perinatal medical history) [24,27].

### 2.7 Primary outcomes

#### 2.7.1 Neurodevelopmental outcomes.

The validated screening tool for Social Responsiveness Scale (SRS-1) as a screening tool for autism spectrum disorders [49,50] and the KINDL quality of life questionnaire [51] will be used.

#### 2.7.2 Cardio-metabolic outcomes.

Waist circumference will be measured, and BMI age-and sex-standardized z-scores will be calculated based on the measurements of weight and height. Systolic and diastolic blood pressure will be measured using automatic upper arm sphygmomanometers.

#### 2.7.3 Respiratory outcomes.

The International Study of Asthma and Allergies in Childhood (ISAAC) questionnaires will be used to assess the prevalence and severity of asthma and allergic disease in children [52].

### 2.8 Statistical considerations

#### 2.8.1 Sample size calculation.

Estimating the sample size in this type of study (child cohort exposome study) presents several challenges, due to the need to account for multiple factors, including the repeated measures design, the analytical variability, the multiplicity of exposures, the correction for multiple testing, and the minimum detectable concentration difference [53,54]. A sample size of 300 children per country was calculated to permit detection of standardized differences of approximately 0.3 over six assessment periods, with 80% power, a significance level of 0.05, and 10% annual attrition. This is expected to yield a final analytic sample of approximately 200 children per country, retained over the minimum of 6 years study duration.

Our sample size is informed by previous exposome and multi-omics studies, such as the HELIX subcohort with 200 mother-child pairs from each of the 6 cohorts [55], demonstrating that cohorts of this size can provide sufficient power to detect moderate associations after multiple testing correction. Moreover, a key strength of our study is the longitudinal design, with five follow-up assessments, allowing each participant to contribute up to six repeated measurements (baseline and five follow-ups) in three country-sites, adding up to a total of 3600 samples. This increases the number of observations available for analysis and enhances the ability to detect associations in multi-omics analyses.

#### 2.8.2 Indicative statistical analyses.

For the characterization of the spatiotemporal variability of the exposomic profiles, descriptive statistics will be used, including the mean, median, standard deviation, and interquartile range. To visualize the variation in high-dimensional exposome data, principal component analysis (PCA) will be used and to identify patterns in exposure profiles, clustering techniques will be employed [56,57]. Furthermore, in order to model children’s exposomic profiling over space and time, linear mixed models (LMMs), generalized linear models, and Bayesian hierarchical models will be applied, as appropriate.

In order to estimate the effect of single exposures on early-life biological markers (such as inflammation and oxidative stress), multivariable regression (linear/logistic/mixed) models will be used, adjusting for confounders, such as exposome-wide association models (ExWAS) [58]. For the exploration of multiple exposures and outcomes association, variable selection algorithms including deletion-substitution-addition (DSA), elastic net (ENET), Bayesian kernel machine regression (BKMR), lagged kernel machine regression (LKMR), hierarchical clustering on principal component, partial least squares-discriminant analysis (PLS-DA) will be used, as needed [56,59].

Weighted quantile sum (WQS) regression will be applied to assess cumulative mixture effects [56]. Structural equation modeling (SEM) and causal mediation analysis will be used to explore whether biomarkers of effect mediate the pathway from exposures to health outcomes [53]. Latent class analysis (LCA) will be applied to group children into exposure and effect clusters [54]. Metabolomics will be used as an intermediate layer between exposures and outcomes with pathway analysis used for transcriptomics and biological enrichment analysis for proteomics.

To generate NCD risk prediction groups, machine learning algorithms, such as random forests, XGBoost, and neural networks will be used, while LASSO will be used for feature selection [56,60]. Unsupervised clustering, like k-means, will be used to define NCD risk groups. Targeted maximum likelihood estimation could also be used to estimate causal effects of modifiable exposures on predicted NCD risk groups [61].

To control overfitting and minimize false-positive findings in high-dimensional exposomic data, a multi-layered statistical strategy will be implemented. Data pre-processing will involve applying strict filters to exclude variables with high homogeneity (>90%) or very high missingness (>70%) and to retain only one exposure from variable pairs with very high correlation (r > 0.9). Dimensionality reduction techniques, such as Principal Component Analysis (PCA) and Partial Least Squares (PLS), will be used to concentrate variance into a smaller number of uncorrelated factors. Furthermore, penalized regression methods including Elastic Net (ENET) and LASSO will be employed for automated feature selection. Methods like PLS and DSA using 5-fold or repeated cross-validation will be employed to calibrate tuning parameters and select models that minimize the Root Mean Square Error (RMSE). Where possible, models will be evaluated using independent data from follow-up assessments to assess their robustness and generalizability. The Benjamini-Hochberg procedure (FDR adjustment) will be used, as appropriate, for multiple comparisons control [62].

Models will adjust for confounders selected using directed acrylic graph (DAG)-informed approaches, literature review and data availability.

#### 2.8.3 Missing data and attrition.

For handling missing data, imputation techniques, such as MICE and missForest, will be utilized [63]. Variables with more than 70% missing data will be excluded from the analyses.

We will address differential attrition and non-random loss to follow-up using descriptive analyses that will compare baseline characteristics of participants who remain in the study with those lost to follow-up, including demographic, socioeconomic and key exposure variables. This will help identify potential patterns of differential attrition. Inverse probability weighting (IPW) will be applied to reduce bias due to non-random loss to follow-up. Probability weights will be estimated using logistic regression models, predicting the likelihood of remaining in the study based on baseline covariates and relevant exposures. These weights will then be incorporated into regression analyses so that participants who are underrepresented due to attrition contribute proportionally more to the estimation of associations [64]. Sensitivity analyses will also be conducted to assess the robustness of results under different assumptions about missing data mechanisms, such as complete-case analyses and weighted analyses.

#### 2.8.4 Federated data analysis using a Common Data Model.

To exploit the multi-country site focus of CHILDREN_FIRST, all data collection methods and study instruments have been *a priori* agreed upon by all participating countries to facilitate federated data processing. To this extent, the reference model of Observational Medical Outcomes Partnership – Common Data Model (OMOP-CDM), maintained and developed as part of the Observational Health Data Sciences and Informatics (OHDSI) initiative, will be used [65]. OHDSI is an open initiative to facilitate the analysis of real-world data via observational studies. On top of this reference data model, various software tools and methodological approaches have been developed and used extensively (statistical analysis approaches, reference dictionaries) to support the execution of observational studies. The European Health Data & Evidence Network (EHDEN) is a European initiative which aims to implement the OHDSI platform and set up a network of partners in Europe [66]. Notably, EHDEN has (so far) attracted more than 180 data partners across Europe, significantly contributing to the setup of the global OHDSI network. Thus, OMOP-CDM has been selected to be used by the CHILDREN_FIRST consortium as it can be considered the de facto standard for real-world data analysis in Europe.

Practically, this means that all CHILDREN_FIRST cohort sites will convert their data to OMOP-CDM. This will allow the formulation of a research question based on the reference CDM, which can then be distributed to each partner site following a “federated” paradigm of data analysis. Each site executes the query locally, and the results are aggregated and analyzed centrally. The advantage of this approach is that there is no need to transfer raw personal/sensitive data, thus avoiding legal and ethical challenges, while robust, large-scale observational analyses across the various populations can take place. Furthermore, the use of OMOP-CDM – the most prominent common data model currently being widely used for observational studies – could facilitate the use of standardized analytics and could potentially support the secondary use of the collected data in collaboration with the international scientific community.

#### 2.8.5 Cross-country replication and validation.

All participating country sites will use the same instruments (questionnaires and measurement devices, e.g., sensors, sphygmomanometers, scales, measuring tapes) and SOPs, strengthening cross-country comparability. Also, federated data analysis using OMOP-CDM will be employed, so that sensitive data are not transferred, but instead each site will run analysis scripts on their own data and only aggregated results will be shared centrally. This will allow for the simultaneous replication of analyses across countries. Furthermore, for internal validation, associations will be evaluated using bootstrap or other cross-validation methods, within each country’s site. For external validation, results will be compared between countries.

### 2.9 Feasibility activities

The feasibility activities aim to provide essential insights that can enable the optimization of recruitment procedures, logistical organization, participant burden, and long-term retention strategies for the main study. In Cyprus, five focus groups with parents of first grade primary school children, teachers and headmasters, as well as a feasibility study with about 20 participants in one school took place in 2023. In Greece, four focus groups with parents of first grade primary school children took place in 2024. The feasibility activities findings highlighted ways to improve recruitment and stakeholder engagement, including optimal timing for meetings with headmasters and teachers, and modifications to recruitment materials’ content and language. They also helped identify opportunities to reduce participant burden, such as, ways to reduce questionnaire completion time and to remind parents to complete the questionnaires. Moreover, these activities informed participant retention strategies for the annual follow-ups, including personalizing results, providing symbolic gifts, and publishing newsletters. Finally, the feasibility study in Cyprus showed the need for several logistical adjustments to streamline participation – for example, the simplification of the interest form, the timing of the yearly assessments and data collection, and improving practical aspects of biospecimen collection, including the time window for the delivery of samples from schools to the biobanks facilities.

### 2.10 Data management

Data use is governed by the CHILDREN_FIRST Data Access Committee (DAC) and follows the EU GDPR data sharing policy and governed by sample access requirements for biobanks. A data management plan will be developed following the principles of Open Access by the European Commission. The plan will address the issues of data cleaning, integration, assessment, and sharing including future publications guidelines. It will be aligned with the GDPR 2016/679 regulation for privacy protection and confidentiality. The data management plan, which will be developed during the project’s planning phase, prior to the initiation of data collection, will represent a mutual understanding between all the project collaborators and will be regularly updated as the project evolves.

Standard Operating Procedures (SOPs) for all study-related activities such as recruitment procedures, field and questionnaire data collection and follow-up assessments, will be developed before the study commences. All study instruments (questionnaires, measurement devices, e.g., sensors, sphygmomanometers, scales, measuring tapes) will be piloted prior to the main study initiation. All SOPs will be documented in a General Survey Manual and will be finalized after their testing during the feasibility phase of the study. To ensure compliance with SOPs, training of all involved collaborators will take place.

Compliance with good scientific practice in general and the agreed SOPs in particular will be ensured by regular reporting schemes. Quality control and assurance will encompass documentation and validation of databases as well as software routines for data warehousing and analysis. Training of project team members will take place for different activities of the study: field/data collection activities, effective engagement with participants, and data management.

Operational procedures will be explicitly defined in the data management plan to enhance transparency and replicability across participating sites. A pre-specified analytical plan will guide analyses, specifying exposure domains, confounder adjustment strategies, and outcome assessments. Study implementation will follow a phased approach, including preparation activities, such as questionnaire standardization and translation, SOPs development and staff training, feasibility activities, schools randomized selection, participant recruitment, baseline data collection, follow-up assessments, and federated data analysis. Data governance will follow established international data protection standards, with individual-level data securely stored at each site and managed according to local regulatory requirements. Analyses will be conducted using a federated framework with harmonized analytical scripts executed locally, and only aggregated results will be shared for pooled interpretation, ensuring both data privacy and methodological consistency across countries.

An electronic toolbox-hub will be developed following the establishment of the study, and it will comprise various interfaces tailored to different user groups: (1) Internal: Research team members will have password-protected access to study data and metadata. All data will be de-identified and pseudo-anonymized to ensure participants’ confidentiality. (2) Parental: Parents will have password-protected access to their child’s individual health and other data. This interface will include personalized reports on biomarker levels accompanied by interpretative information, such as reference ranges (where available), comparisons with age-matched population averages, and relevant recommendations. (3) Aggregated: Policy makers and school authorities will have access to aggregated data (with the city as the unit of reference). This will include summaries of various biomarker levels and health-related outcomes measured across the study population. (4) Public: The public will have access to scientific findings in the form of flyers and infographics. (5) Open access: Datasets will be available upon request after the submission and evaluation of a data application by interested research teams.

### 2.11 Ethics and dissemination

The study protocol has been approved by the Cyprus National Bioethics Committee (EEBK/EP/2022/68) and the Cyprus Ministry of Education, Sports and Youth (07.15.005.011.001).

All study procedures will be conducted in accordance with the principles of the World Medical Association Declaration of Helsinki, and the study will have local ethics approval, abiding by the ethical standards of each participating country. Written informed consent will be available in each country’s official language and in English, and it will be obtained from parents/guardians. Verbal assent will be required by each child before any type of assessment. Parents have the right to withdraw approval for their child’s participation at any stage, for any reason, without having to give any explanations and without any consequences for the participant. The study does not involve any risks, since the sampling is non-invasive. All data will be treated with strict confidentiality. A unique identification code will be assigned to each participating child and will be used in questionnaires, biological samples, and databases.

Study results will be disseminated at regional and international conferences and in peer-reviewed journals. Different types of results (personalized, aggregated) will be available to distinct user groups, i.e., parents and schools, respectively, through the electronic toolbox-hub (see the Data management section). Aggregated cohort-level results will be communicated annually through newsletters. Until the electronic toolbox-hub is fully developed, individualized reports will be delivered to parents on an annual basis through a secure, password-protected system. These reports will include detailed interpretations of the assessed parameters and personalized recommendations tailored to the child’s data.

Results that will be returned to the participants will include those for biomarkers with established reference ranges and clinically recognized cut-offs, using clear and appropriate language. The selection of biomarkers will be finalized in collaboration with the Participant Advisory Committee and may be updated in future follow-ups to reflect clinical changes as well as feedback from parents. All biomarker assessments will be reviewed to identify values outside established reference ranges. Out-of-range or abnormal findings will be flagged according to pre-specified thresholds, based on clinically validated cut-offs and age- and sex-specific norms, where available. The research team will verify these results, considering potential measurement errors or data entry mistakes. A research team member will contact promptly the parents/guardians by telephone to inform them of the out-of-range finding, providing clear, non-alarming explanations and allowing participants to ask questions before the formal report is issued. The recommendation will be to share the results with their paediatrician and, where relevant, to repeat the test/analysis.

### 2.12 Patient and public involvement statement

An integral part for the success of CHILDREN_FIRST is the active engagement of participants, their families, and the school personnel. To facilitate this, a multifaceted engagement approach will be applied. Prior to study initiation, focus groups with parents, teachers, and headmasters will be conducted to discuss the study instruments, the ethical and security issues including the risks and benefits of the CHILDREN FIRST study protocol and gather their perspectives on strategies for maintaining participation throughout follow-up assessments. A feasibility study with a small number of participants will take place to assess response rates and key study procedures, including recruitment, data collection, and the transfer and storage of samples.

An Advisory Board, comprising experts in environmental epidemiology, biomedical sciences, exposure science, the exposome field, and the entrepreneurship sector will be established to support the study. The Advisory Board will play a critical role in monitoring and providing constructive input on the study’s progress, offering recommendations for addressing emerging challenges, advising on strategic planning, and contributing insights to enhance the study’s societal impact.

A Participant Advisory Committee, composed of interested parents of children participating in the study, will be assembled to provide ongoing input. The committee will meet regularly to discuss various topics, including data collection procedures, reports of individualized results, and the incorporation of participant feedback.

Research staff will be trained to have an effective dialogue with children and their parents/guardians so that they can help promote participants’ empowerment and ensure long-term participant engagement. Regular communication will be maintained through personal interactions, phone calls and emails. A dedicated hotline at each site will be available for parents to ask questions or share concerns.

The expected benefit for participants is that they will receive annual personalized reports containing selected data related to their children’s assessments. School headmasters will receive annual reports on the schools’ air and water quality and will be encouraged to share these results with parents and school boards. Aggregated results on children’s environmental and health indicators will be shared periodically with parents and relevant stakeholders (e.g., media, policymakers). Moreover, newsletters will be distributed to parents annually, providing updates on the study’s progress and offering opportunities for feedback. All participating children will also receive a symbolic gift for each annual assessment.

### 2.13 Timeline

**Fig 3:**
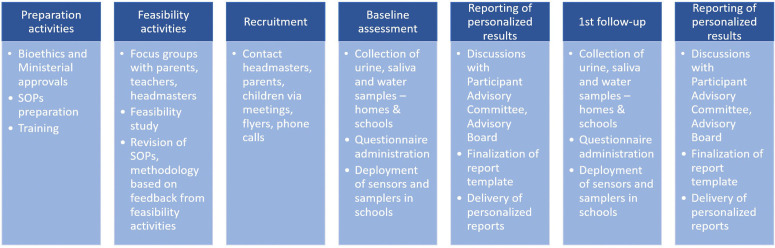
CHILDREN_FIRST project timeline. Subsequent follow-up assessments (2nd–5th) are not shown; these will be conducted annually, following the same schedule as the first follow-up assessment.

An indicative timeline of the CHILDREN_FIRST main activities is shown in Fig 3. In Cyprus, participant recruitment took place between October 2023 and January 2024 and data collection for the baseline assessment was conducted from January 2024 to June 2024. In Greece, feasibility activities took place in 2024. In Greece and Albania, the participant recruitment is expected to be completed by 2027. Federated results are expected to be available by 2034.

## 3 Discussion

CHILDREN_FIRST is unique in its scope and design, representing the first *a priori* harmonized, exposome-based children’s cohort that will conduct annual follow-ups across three Mediterranean countries during the critical windows of vulnerability (6–11 years of age). The CHILDREN_FIRST study integrates the exposome concept and personalized prevention approaches to examine multiple environmental exposures and health outcomes over time. The anticipated impact can be outlined under two key pillars: Methodological novelties and generation of novel knowledge.

### 3.1 Methodological novelties

#### 3.1.1 First exposome-based children’s prospective cohort in the Mediterranean region.

CHILDREN_FIRST will be the first exposome-based children’s longitudinal study in the Mediterranean countries of Cyprus, Greece and Albania. Unlike most pregnancy-birth cohorts, CHILDREN_FIRST targets ages 6 to 11 years upon child’s entry to primary school, which is a relatively understudied, yet critical developmental period of life [8]. This study will conduct annual assessments beginning in first grade all the way through the final year of primary school, collecting a maximum of six repeated measurements per child over six years. The integration of multi-omics platforms will be key in disentangling the gene expression patterns and the downstream biological processes that govern children’s growth and development trajectories in the co-occurrence of a suite of environmental stressors.

#### 3.1.2 Development of precision prevention-based datasets for informed policy making and development of health interventions.

The collection of annual cohort data and the generation of NCD-stratified disease risk groups by their exposomic profiles during a critical window of susceptibility will generate robust scientific evidence to support the development of evidence-based guidelines and interventions. These will be disseminated to relevant policymakers, including the Ministries of Health and Education in all participating countries, the WHO/UNESCO/UNICEF health-promoting schools unit, and beyond. By implementing such measures, the study aims to contribute to reducing the future incidence of NCDs, hence lowering governmental healthcare expenditures and broader societal costs.

#### 3.1.3 Long-term cohort infrastructure for future exposomic research and collaboration.

The CHILDREN_FIRST cohort will produce spatio-temporal data on child health of high-quality, complemented by biospecimen collection and storage to biobanks and an electronic toolbox-hub. This infrastructure will not only support the current study objectives but will also serve as a long-term resource for hypothesis generation and testing and it will facilitate collaboration with local and EU institutions as well as with other cohort studies.

#### 3.1.4 Emphasis on the school setting.

The CHILDREN_FIRST cohort has intentionally focused on the school setting to inform, recruit, collect data and samples, and inform parents and many stakeholders as part of the cohort’s related activities. This is a cost-efficient approach to engaging stakeholders in the children’s health prevention programs towards achieving healthy growth and development for all children, leaving no one behind.

### 3.2 Generation of novel knowledge

#### 3.2.1 Understanding environmental impacts on early disease development.

CHILDREN_FIRST aims to advance the understanding of how multiple environmental exposures affect the development of diseases and the progression of early-stage disease markers during the vulnerable developmental window of ages 6–11 years.

#### 3.2.2 Uncovering the temporal biodynamics of early-stage disease markers.

The collection of a high number of repeated measurements during a critical life stage will allow detailed investigation of the temporal dynamics of disease processes. Particular emphasis will be given to early-stage disease markers, such as biomarkers of oxidative stress, inflammation, and omics-based signatures.

While changes in anthropometric indicators, such as BMI, are expected within one-year intervals, data on the temporal trend of obesogenic molecular markers in this age group remain scarce. The CHILDREN_FIRST study will be instrumental in identifying key windows of susceptibility and the timing of potential interventions.

For example, existing evidence suggests that interventions as early as at 6 months old can significantly improve developmental trajectories in children at risk for autism spectrum disorders [67]. Similarly, global data indicate that the prevalence of overweight among children aged 5–19 years (18% in 2016) is considerably higher than in children under five (5.6% in 2019) [68]. Assessing how BMI and related biomarkers evolve during primary school years could provide critical insights into the metabolic disease processes. Similar hypotheses could also extend to other outcomes, such as cardio-metabolic and neurodevelopmental.

#### 3.2.3 Identification of novel early-stage biomarkers.

The application of multiple omics platforms, such as metabolomics, will facilitate the comprehensive profiling of the children’s exposome. Multi-omics platforms, such as genomics, transcriptomics, epigenomics, microbiomics, proteomics and metabolomics may be used as intermediaries, i.e., mediators between main exposomic variables and the key outcomes or end points of disease. Machine learning algorithms will explore associations between omics signatures and both exposure profiles and health outcomes, potentially leading to the identification of novel biomarkers that serve as early indicators of chronic disease risk or lead to NCD risk prediction groups with distinct exposomic profiles.

### 3.3 What does this prospective children’s exposomics cohort study add?

CHILDREN_FIRST introduces several innovative contributions to biomedical and environmental health research for children, particularly within the context of the broader Mediterranean region and its heterogeneous populations:

#### 3.3.1 Prospective health data collection for middle childhood.

The cohort will collect longitudinal population-level health data specifically for children aged 6–11 years in Cyprus, Greece, and Albania.

#### 3.3.2 Spatio-temporal characterization of the child exposome.

The study will generate novel insights into the spatial and temporal variability of exposome data within the child population in these three countries, improving the understanding of how environmental exposures differ across time and geographical area.

#### 3.3.3 High-frequency, repeated exposomic measurements with non-invasive biomarkers of exposure/effect.

By conducting six annual assessment points over six consecutive years, this study enables repeated data collection within a relatively short timeframe, an approach that enhances the ability to detect temporal trends and exposomic trajectories that would prospectively frame up children’s growth and development curves.

#### 3.3.4 Assessment of novel environmental stressors.

The cohort includes evaluation of emerging and underexplored exposures, such as artificial light at night (ALAN) or microplastics or PFAS, among others, and investigates their potential health impact on children’s growth and development.

#### 3.3.5 Integration of environmental and health profiles using exposomics.

CHILDREN_FIRST will apply an exposome-based framework to integrate environmental exposure data with health outcomes, leveraging advanced tools for comprehensive profiling of the environment-health interface.

#### 3.3.6 Stakeholder-specific data dissemination via an electronic toolbox-hub.

A secure digital platform (toolbox-hub) will facilitate access to study findings, with tailored interfaces for different stakeholders: (1) Parents will receive personalized reports for their child, including biomarker interpretations and recommendations. (2) Policymakers and school authorities will be able to access aggregated, city-level data to inform public health strategies. (3) Researchers will have access to de-identified datasets for further analysis.

#### 3.3.7 Adoption of common data models.

Multi-country cohort data will be processed using the OMOP-CDM, promoting data interoperability, facilitating collaboration and enhancing the potential for data reuse in future research, including secondary use in future studies.

### 3.4 Limitations and strengths

Unlike most birth cohorts that collect data during pregnancy and beyond, our study begins recruitment at the start of primary school. However, data on birth and perinatal periods will be retrospectively gathered through validated questionnaires completed by parents, which may introduce recall bias. The sample size may be relatively modest (n = 200–300 per site), but the longitudinal design – featuring six repeated measurements over six years – will yield approximately 1200–1800 data points per cohort site, providing rich datasets with enhanced statistical power. On the other hand, CHILDREN_FIRST will be the first longitudinal, exposome-based and a priori harmonized children's multi-country site cohort study with annual follow-ups in Mediterranean countries – Cyprus, Greece, and Albania – using the same study instruments/tools with the prospect to include other Mediterranean sites in the future. Study procedures, such as recruitment, assessments and reporting back, will be centered on the school setting, transforming schools into health-promoting schools (HPS), based on the WHO/UNESCO/UNICEF HPS paradigm towards integrating health into all aspects of school life. The high number of repeated measurements during a pre-defined critical life stage of vulnerability (6–11 years), with baseline at entry in primary school (around 6 years of age) will allow for delineating the temporal dynamics of chronic disease processes as impacted by children’s exposomics trajectories during this critical developmental window. The spatiotemporal profiling of children’s exposomes will provide the basis for exploration of associations between multi-omics signatures, biomarkers of exposure/effect profiles and health outcomes, potentially identifying novel non-genetic markers as early indicators of chronic disease risk. The collection of annual cohort data and the generation of NCD-stratified disease risk groups by their exposomic profiles during a critical window of susceptibility will generate robust scientific data to inform evidence-based guidelines and non-pharmacological health interventions.

### 3.5 Future directions and implications

CHILDREN_FIRST is an ambitious initiative that will establish a long-term prospective cohort infrastructure for biomedical research on children’s health within the Mediterranean region linked with established biobanks, such as the biobank.cy. It will be key in framing and advancing the epidemiological surveillance of chronic diseases during childhood, in collaboration with relevant institutions, public health bodies and universities. This study is aligned with the European Commission’s principles of personalized medicine. The cohort findings will systematically feed into the evaluation and design of chronic disease prevention programs. Expected results would inform evidence-based policy making and the development of health interventions for reducing the risk of NCDs. The effective implementation of such disease prevention and control programs fed by CHILDREN_FIRST cohort results would generate substantial reductions in social costs of NCDs with longer-term benefits for the health sector, the quality of life, and wellbeing.

Although the CHILDREN_FIRST cohort is established in Mediterranean populations, many of the biological and environmental mechanisms under investigation are likely relevant across different settings. However, differences in environmental exposures, dietary patterns, socioeconomic conditions, cultural behaviors, and healthcare systems may influence both exposure distributions and health outcomes. Therefore, while the study is expected to provide valuable insights into different environmental determinants of health, caution is warranted when extrapolating findings to non-Mediterranean populations. Replication of key findings in exposome-based cohorts from other geographic regions and with different exposure patterns will be important to further assess the external validity and generalizability of the results.

## Supporting information

S1 File Methodological details. (DOCX)

S2 File SPIROS checklist. (DOCX)

## References

[1] Bloom DE, Cafiero ET, Jané-Llopis E, Abrahams-Gessel S, Bloom LR, Fathima S. The Global Economic Burden of Noncommunicable Diseases. Geneva: World Economic Forum; 2011.

[2] Armocida B, Monasta L, Sawyer S, Bustreo F, Segafredo G, Castelpietra G, et al. Burden of non-communicable diseases among adolescents aged 10–24 years in the EU, 1990–2019: a systematic analysis of the Global Burden of Diseases Study 2019. Lancet Child Adolesc Health. 2022;6:367–83. doi: 10.1016/s2352-4642(22)00073-6 35339209 PMC9090900

[3] GBD 2019 Cancer Risk Factors Collaborators. The global burden of cancer attributable to risk factors, 2010-19: a systematic analysis for the Global Burden of Disease Study 2019. Lancet Lond Engl. 2022;400:563–91. doi: 10.1016/S0140-6736(22)01438-6 PMC9395583 35988567

[4] World Health Organization. Global action plan for the prevention and control of noncommunicable diseases 2013-2020. Geneva: World Health Organization; 2013. Available: https://apps.who.int/iris/handle/10665/94384

[5] UN General Assembly. Transforming our world: the 2030 Agenda for Sustainable Development. 2015. Report No.: A/RES/70/1. Available: https://sdgs.un.org/sites/default/files/publications/21252030%20Agenda%20for%20Sustainable%20Development%20web.pdf

[6] World Health Organization. Thirteenth General Programme of Work, 2019–2023. Geneva: World Health Organization; 2018. Available: http://www.who.int/about/what-wedo/gpw-thirteen-consultation/en/

[7] Landrigan PJ, Kimmel CA, Correa A, Eskenazi B. Children’s health and the environment: public health issues and challenges for risk assessment. Environ Health Perspect. 2004;112(2):257–65. doi: 10.1289/ehp.6115 14754581 PMC1241836

[8] Cohen Hubal EA, de Wet T, Du Toit L, Firestone MP, Ruchirawat M, van Engelen J, et al. Identifying important life stages for monitoring and assessing risks from exposures to environmental contaminants: results of a World Health Organization review. Regul Toxicol Pharmacol. 2014;69(1):113–24. doi: 10.1016/j.yrtph.2013.09.008 24099754 PMC5355211

[9] Ward ZJ, Long MW, Resch SC, Giles CM, Cradock AL, Gortmaker SL. Simulation of Growth Trajectories of Childhood Obesity into Adulthood. N Engl J Med. 2017;377(22):2145–53. doi: 10.1056/NEJMoa1703860 29171811 PMC9036858

[10] Bui DS, Lodge CJ, Burgess JA, Lowe AJ, Perret J, Bui MQ, et al. Childhood predictors of lung function trajectories and future COPD risk: a prospective cohort study from the first to the sixth decade of life. Lancet Respir Med. 2018;6(7):535–44. doi: 10.1016/S2213-2600(18)30100-0 29628376

[11] European Parliament. Directorate General for Parliamentary Research Services. Human exposome research: potentials, limitations and public policy implications. LU: Publications Office; 2025. Available: https://data.europa.eu/doi/10.2861/5600247

[12] Wild CP. Complementing the genome with an “exposome”: the outstanding challenge of environmental exposure measurement in molecular epidemiology. Cancer Epidemiology, Biomarkers & Prevention. 2005:1847–50. doi: 10.1158/1055-9965.EPI-05-0456 16103423

[13] Wild CP. The exposome: from concept to utility. Int J Epidemiol. 2012;41(1):24–32. doi: 10.1093/ije/dyr236 22296988

[14] Haddad N, Andrianou XD, Makris KC. A scoping review on the characteristics of human exposome studies. Curr Pollut Rep. 2019;5:378–93. doi: 10.1007/s40726-019-00130-7

[15] Willett WC. Balancing life-style and genomics research for disease prevention. Science. 2002;296(5568):695–8. doi: 10.1126/science.1071055 11976443

[16] European Commission. Personalised Medicine. 2020. Available: https://research-and-innovation.ec.europa.eu/research-area/health/personalised-medicine_en

[17] Gambhir SS, Ge TJ, Vermesh O, Spitler R. Toward achieving precision health. Sci Transl Med. 2018;10(430):eaao3612. doi: 10.1126/scitranslmed.aao3612 29491186 PMC5985668

[18] Vrijheid M, Slama R, Robinson O, Chatzi L, Coen M, van den Hazel P, et al. The human early-life exposome (HELIX): project rationale and design. Environ Health Perspect. 2014;122(6):535–44. doi: 10.1289/ehp.1307204 24610234 PMC4048258

[19] Maitre L, de Bont J, Casas M, Robinson O, Aasvang GM, Agier L, et al. Human Early Life Exposome (HELIX) study: a European population-based exposome cohort. BMJ Open. 2018;8(9):e021311. doi: 10.1136/bmjopen-2017-021311 30206078 PMC6144482

[20] Jacobson LP, Parker CB, Cella D, Mroczek DK, Lester BM, program collaborators for Environmental influences on Child Health Outcomes. Approaches to protocol standardization and data harmonization in the ECHO-wide cohort study. Pediatr Res. 2024;95:1726–33. doi: 10.1038/s41390-024-03039-0 38365871 PMC11245389

[21] Hughes RA, Tilling K, Lawlor DA. Combining Longitudinal Data From Different Cohorts to Examine the Life-Course Trajectory. Am J Epidemiol. 2021;190(12):2680–9. doi: 10.1093/aje/kwab190 34215868 PMC8634562

[22] Google. NotebookLM. 2024. Available: https://notebooklm.google/

[23] Mahajan R, Burza S, Bouter LM, Sijtsma K, Knottnerus A, Kleijnen J, et al. Standardized Protocol Items Recommendations for Observational Studies (SPIROS) for Observational Study Protocol Reporting Guidelines: Protocol for a Delphi Study. JMIR Res Protoc. 2020;9(10):e17864. doi: 10.2196/17864 33084592 PMC7641775

[24] EURO-URHIS 2. European Urban Health Indicators Part Two: Using indicators to inform policy. 2014 Dec. Available: https://cordis.europa.eu/project/id/223711/reporting

[25] Statistical Service of Cyprus. European Health Interview Survey. 2016 Nov. Available: https://library.cystat.gov.cy/Documents/Questionnaires/EUROPEAN_HEALTH_SURVEY-2014-EN.pdf

[26] Statistical Service of Cyprus. Survey on Income and Living Conditions of Households 2022. Feb 2022. Available: https://www.cystat.gov.cy/en/QuestionnaireList?s=44

[27] González-Alzaga B, Hernández AF, Kim Pack L, Iavicoli I, Tolonen H, Santonen T, et al. The questionnaire design process in the European Human Biomonitoring Initiative (HBM4EU). Environ Int. 2022;160:107071. doi: 10.1016/j.envint.2021.107071 34979351

[28] Charisiadis P, Andra SS, Makris KC, Christophi CA, Skarlatos D, Vamvakousis V, et al. Spatial and seasonal variability of tap water disinfection by-products within distribution pipe networks. Sci Total Environ. 2015;506–507:26–35. doi: 10.1016/j.scitotenv.2014.10.071 25460936

[29] Nomura H, Ueyama J, Kondo T, Saito I, Murata K, Iwata T, et al. Quantitation of neonicotinoid metabolites in human urine using GC-MS. J Chromatogr B Analyt Technol Biomed Life Sci. 2013;941:109–15. doi: 10.1016/j.jchromb.2013.10.012 24189204

[30] Leng G, Gries W. Determination of Pyrethroids in Blood Plasma and Pyrethroid/Pyrethrin Metabolites in Urine by Gas Chromatography-Mass Spectrometry and High-Resolution GC-MS BT - Pesticide Protocols. In: Martínez Vidal JL, Frenich AG, editors. Totowa, NJ: Humana Press; 2006. pp. 17–33. doi: 10.1385/1-59259-929-X:017

[31] CDC. Laboratory Procedure Manual. Matrix: Urine. Method: Urine Multi-Element ICP-DRC-MS Renamed from Inductively Coupled Plasma-Mass Spectrometry (ICP-DRC-MS). Method No: 3018.3 (15 element panel) and 3018A.2 (total arsenic). Centers for Disease Control and Prevention. Inorganic Radionuclides and Toxicology, Division of Laboratory Sciences, National Center for Environmental Health; 2012. p. 1–48. Available: https://www.cdc.gov/nchs/data/nhanes/nhanes_11_12/uhm_g_met_heavy_metals.pdf

[32] Kalyvas H, Andra SS, Charisiadis P, Karaolis C, Makris KC. Influence of household cleaning practices on the magnitude and variability of urinary monochlorinated bisphenol A. Sci Total Environ. 2014;490:254–61. doi: 10.1016/j.scitotenv.2014.04.072 24858223

[33] ISGlobal. HELIX Project: Data Inventory (Subcohort). 2014 [cited 20 Jan 2023]. Available: http://www.projecthelix.eu/data-inventory

[34] Baloch RM, Maesano CN, Christoffersen J, Banerjee S, Gabriel M, Csobod É, et al. Indoor air pollution, physical and comfort parameters related to schoolchildren’s health: Data from the European SINPHONIE study. Sci Total Environ. 2020;739:139870. doi: 10.1016/j.scitotenv.2020.139870 32544681

[35] Garcia-Saenz A, Sánchez de Miguel A, Espinosa A, Valentin A, Aragonés N, Llorca J, et al. Evaluating the Association between Artificial Light-at-Night Exposure and Breast and Prostate Cancer Risk in Spain (MCC-Spain Study). Environ Health Perspect. 2018;126(4):047011. doi: 10.1289/EHP1837 29687979 PMC6071739

[36] Makris KC, Konstantinou C, Andrianou XD, Charisiadis P, Kyriacou A, Gribble MO, et al. A cluster-randomized crossover trial of organic diet impact on biomarkers of exposure to pesticides and biomarkers of oxidative stress/inflammation in primary school children. PLoS One. 2019;14(9):e0219420. doi: 10.1371/journal.pone.0219420 31483785 PMC6726134

[37] Klakk H, Wester CT, Olesen LG, Rasmussen MG, Kristensen PL, Pedersen J, et al. The development of a questionnaire to assess leisure time screen-based media use and its proximal correlates in children (SCREENS-Q). BMC Public Health. 2020;20(1):664. doi: 10.1186/s12889-020-08810-6 32397984 PMC7216486

[38] López-Gajardo MA, Leo FM, Sánchez-Miguel PA, López-Gajardo D, Soulas C, Tapia-Serrano MA. KIDMED 2.0, An update of the KIDMED questionnaire: Evaluation of the psychometric properties in youth. Front Nutr. 2022;9:945721. doi: 10.3389/fnut.2022.945721 36424923 PMC9679638

[39] Bruni O, Ottaviano S, Guidetti V, Romoli M, Innocenzi M, Cortesi F, et al. The Sleep Disturbance Scale for Children (SDSC). Construction and validation of an instrument to evaluate sleep disturbances in childhood and adolescence. J Sleep Res. 1996;5(4):251–61. doi: 10.1111/j.1365-2869.1996.00251.x 9065877

[40] Foraster M, Künzli N, Aguilera I, Rivera M, Agis D, Vila J, et al. High blood pressure and long-term exposure to indoor noise and air pollution from road traffic. Environ Health Perspect. 2014;122(11):1193–200. doi: 10.1289/ehp.1307156 25003348 PMC4216159

[41] Werner H, Lebourgeois MK, Geiger A, Jenni OG. Assessment of chronotype in four- to eleven-year-old children: reliability and validity of the Children’s Chronotype Questionnaire (CCTQ). Chronobiol Int. 2009;26(5):992–1014. doi: 10.1080/07420520903044505 19637055 PMC2988284

[42] Chan ECY, Pasikanti KK, Nicholson JK. Global urinary metabolic profiling procedures using gas chromatography-mass spectrometry. Nat Protoc. 2011;6(10):1483–99. doi: 10.1038/nprot.2011.375 21959233

[43] Palanisamy V, Wong DT. Transcriptomic Analyses of Saliva. In: Seymour GJ, Cullinan MP, Heng NCK, editors. Oral Biology. Totowa, NJ: Humana Press; 2010. p. 43–51. doi: 10.1007/978-1-60761-820-1_4 PMC3171800 20717777

[44] Joshi N, Garapati K, Ghose V, Kandasamy RK, Pandey A. Recent progress in mass spectrometry-based urinary proteomics. Clin Proteomics. 2024;21(1):14. doi: 10.1186/s12014-024-09462-z 38389064 PMC10885485

[45] Angerer J, editors Hartwig A. The MAK-Collection for Occupational Health and Safety. In: Angerer J, Hartwig A, editors. The MAK-Collection for Occupational Health and Safety. Weinheim: Wiley-VCH Verlag GmbH & Co. KGaA; 2010. p. 169–84.

[46] CDC. Urine iodine ICPMS [Renamed from inductively coupled plasma-mass spectrometry (ICP-MS)], ITU004A. 2007 Dec. Available: https://www.cdc.gov/nchs/data/nhanes/nhanes_03_04/l06uio_c_met_urine_iodine_ICPMS.pdf

[47] WHO. Iodine deficiency. 2013. Available: https://www.who.int/data/nutrition/nlis/info/iodine-deficiency

[48] Moon J-Y, Jung H-J, Moon MH, Chung BC, Choi MH. Heat-map visualization of gas chromatography-mass spectrometry based quantitative signatures on steroid metabolism. J Am Soc Mass Spectrom. 2009;20(9):1626–37. doi: 10.1016/j.jasms.2009.04.020 19497759

[49] Constantino JN. The Social Responsiveness Scale. Los Angeles: Western Psychological Services; 2002.

[50] Constantino JN, Gruber CP. Social Responsiveness Scale: Manual. Los Angeles: Western Psychological Services; 2005.

[51] Ravens-Sieberer U, Bullinger M. Assessing health-related quality of life in chronically ill children with the German KINDL: first psychometric and content analytical results. Qual Life Res. 1998;7(5):399–407. doi: 10.1023/a:1008853819715 9691720

[52] Asher W. The International Study of Asthma and Allergies in Childhood (ISAAC). Clin Experimental Allergy. 1998;28(s5):52–66. doi: 10.1046/j.1365-2222.1998.028s5052.x 9988448

[53] Liu SH, Weber ES, Manz KE, McCarthy KJ, Chen Y, Schüffler PJ, et al. Assessing the Impact and Cost-Effectiveness of Exposome Interventions on Alzheimer’s Disease: A Review of Agent-Based Modeling and Other Data Science Methods for Causal Inference. Genes (Basel). 2024;15(11):1457. doi: 10.3390/genes15111457 39596657 PMC11593565

[54] Santaolalla A, Garmo H, Grigoriadis A, Ghuman S, Hammar N, Jungner I, et al. Metabolic profiles to predict long-term cancer and mortality: the use of latent class analysis. BMC Mol Cell Biol. 2019;20(1):28. doi: 10.1186/s12860-019-0210-7 31337337 PMC6651931

[55] Vrijheid M, Maitre L. Building an Early Life Exposome by Integrating Multiple Birth Cohorts: HELIX. In: Dagnino S, Macherone A, editors. Unraveling the Exposome. Cham: Springer International Publishing; 2019. p. 393–404. doi: 10.1007/978-3-319-89321-1_15

[56] Maitre L, Guimbaud J-B, Warembourg C, Güil-Oumrait N, Petrone PM, Chadeau-Hyam M, et al. State-of-the-art methods for exposure-health studies: Results from the exposome data challenge event. Environ Int. 2022;168:107422. doi: 10.1016/j.envint.2022.107422 36058017

[57] Chang L, Ewald J, Hui F, Bayen S, Xia J. A data-centric perspective on exposomics data analysis. Exposome. 2024;4(1). doi: 10.1093/exposome/osae005

[58] Chung MK, House JS, Akhtari FS, Makris KC, Langston MA, Islam KT, et al. Decoding the exposome: data science methodologies and implications in exposome-wide association studies (ExWASs). Exposome. 2024;4(1):osae001. doi: 10.1093/exposome/osae001 38344436 PMC10857773

[59] Warembourg C, Anguita-Ruiz A, Siroux V, Slama R, Vrijheid M, Richiardi L, et al. Statistical Approaches to Study Exposome-Health Associations in the Context of Repeated Exposure Data: A Simulation Study. Environ Sci Technol. 2023;57(43):16232–43. doi: 10.1021/acs.est.3c04805 37844068 PMC10621661

[60] Isola S, Murdaca G, Brunetto S, Zumbo E, Tonacci A, Gangemi S. The Use of Artificial Intelligence to Analyze the Exposome in the Development of Chronic Diseases: A Review of the Current Literature. Informatics. 2024;11(4):86. doi: 10.3390/informatics11040086

[61] Smith MJ, Phillips RV, Luque-Fernandez MA, Maringe C. Application of targeted maximum likelihood estimation in public health and epidemiological studies: a systematic review. Ann Epidemiol. 2023;86:34-48.e28. doi: 10.1016/j.annepidem.2023.06.004 37343734

[62] Chen S-Y, Feng Z, Yi X. A general introduction to adjustment for multiple comparisons. J Thorac Dis. 2017;9(6):1725–9. doi: 10.21037/jtd.2017.05.34 28740688 PMC5506159

[63] Sun Y, Li J, Xu Y, Zhang T, Wang X. Deep learning versus conventional methods for missing data imputation: A review and comparative study. Expert Systems with Applications. 2023;227:120201. doi: 10.1016/j.eswa.2023.120201

[64] Howe CJ, Cole SR, Lau B, Napravnik S, Eron JJ Jr. Selection Bias Due to Loss to Follow Up in Cohort Studies. Epidemiology. 2016;27(1):91–7. doi: 10.1097/ede.0000000000000409 26484424 PMC5008911

[65] Observational Health Data Sciences and Informatics. 2025. Available: https://www.ohdsi.org/

[66] European Health Data & Evidence Network. 2025. Available: https://www.ehden.eu/

[67] Bradshaw J, Steiner AM, Gengoux G, Koegel LK. Feasibility and effectiveness of very early intervention for infants at-risk for autism spectrum disorder: a systematic review. J Autism Dev Disord. 2015;45(3):778–94. doi: 10.1007/s10803-014-2235-2 25218848

[68] World Health Organization. Obesity and overweight. 2021 [cited 27 June 2022]. Available: https://www.who.int/news-room/fact-sheets/detail/obesity-and-overweight

