# Supplementary material for: Designing a children’s health exposomics study protocol: The CHILDREN_FIRST multi-country prospective cohort using multi-omics and personalized prevention approaches
Source: PLoS One. 2026 Apr 27;21(4):e0326641. doi: 10.1371/journal.pone.0326641 (PMC13119864; doi:10.1371/journal.pone.0326641)
Supplement: S1 File — (DOCX) [file pone.0326641.s001.docx]

**Designing a children’s health exposomics study protocol: The CHILDREN_FIRST multi-country prospective cohort using multi-omics and personalized prevention approaches**

S1 File

## Recruitment

Headmasters of randomly selected schools will be contacted, and the study will be explained. Following the headmaster’s agreement, parents/guardians will be invited to meetings that will take place at the school setting (communication with school for suitable time of event for parents). During the meeting with parents/guardians, a short presentation with a Q&A session will take place and the interest/non-interest form will be provided to all parents/guardians of first graders (even if they did not participate in the meeting). Relevant meetings with children and teachers to inform them about the study will also take place at the school setting. After receiving the completed interest forms, the research team will check if eligibility criteria apply and will inform parents accordingly via telephone. A detailed explanation of the study procedures and the consent form parts will take place during telephone communication. Following the parents/guardians signed consent for their child to participate in the study, each child will be informed orally about the assessment by a member of the research team using a simplified preformulated text, to ensure that the child will give verbal assent before any type of assessment.

## Assessment Methods

### Questionnaires

All questionnaires will be completed by parents/guardians, except for the questionnaires on school building and class characteristics, which will be completed by the school headmaster and teachers. Questionnaires will be developed using the REDCap software [1] and will be standardized, translated and back translated, to ensure consistency across study sites. Prior to finalization, the questionnaires will be piloted to assess clarity and estimate completion time, which will be communicated to parents/guardians.

### Sensors

Sensors will be used to measure PM_10_, PM_2.5_, air temperature, relative humidity, CO_2_, and biogenic volatile organic compounds (VOCs). Teams of 2–3 trained researchers will be responsible for the installation/removal of the sensors based on SOPs that will be developed. Sensors will be installed in indoor (classrooms) and outdoor locations of participating schools for at least 1 school week per year.

### Passive samplers

Passive air samplers will be used to measure benzene, toluene, ethylbenzene and xylene (BTEX) and chloroform in indoor and outdoor locations of participating schools for at least 1 school week per year, based on SOP that will be developed.

### Anthropometric measurements

Anthropometric measurements of children (weight, height, and waist circumference) will be conducted by trained researchers at school premises based on standardized methods [2].

### Blood pressure measurements

Systolic and diastolic blood pressure will be measured three times per child, using automatic upper arm sphygmomanometers, based on standardized methods.

### Biological samples

Sterilized polypropylene urine vials along with instructions for sample collection will be provided to children. With their parents’ help, children will collect first morning urine voids at home on a designated date and hand them over to research team members at school. The urine samples will be temporarily stored in a cooler (4°C) until they are transferred to laboratory facilities for long-term storage at -80°C. On the same day, saliva samples will be collected by research team members at school premises using a commercially available kit, following the manufacturer’s instructions.

### Water samples

Tap water samples will be collected from participating schools and children’s houses. The main faucet used for satisfying the water needs of the participating school and home and it is directly connected to the municipality’s water supply will be selected for drinking-water sampling. Before sample collection, the faucet will be externally cleaned with ethanol and water will be allowed to flow freely for approximately 30 seconds.

**Questionnaires list**

There are some exposome variables that will not be captured with questionnaires, but rather with human biomonitoring, satellite data, sensors and passive samplers. Here the questionnaire-based exposome variables are described by exposome domain and their groups of components based on Haddad et al., 2019

Contents

[General external exposome 5](#_Toc127276500)

[Socio-economic status 5](#_Toc127276501)

[Green spaces 5](#_Toc127276502)

[Psychological and mental stress 5](#_Toc127276503)

[Policies and programs 5](#_Toc127276504)

[Specific external exposome 5](#_Toc127276505)

[Noise 5](#_Toc127276506)

[Chemical contaminants 6](#_Toc127276507)

[Occupation 6](#_Toc127276508)

[Lifestyle 6](#_Toc127276509)

[Internal exposome 7](#_Toc127276510)

[Basic characteristics 7](#_Toc127276511)

[Intrinsic properties 7](#_Toc127276512)

[Reproduction-related variables 7](#_Toc127276513)

[Biochemistry and medical indicators 8](#_Toc127276514)

[Additional questions 8](#_Toc127276515)

[Questions relevant for older children (≥ 9 years old) 8](#_Toc127276516)

[References 9](#_Toc127276517)

General external exposome

## Socio-economic status

- Survey on Income and Living Conditions of Households 2022 (selected questions for address, municipality, district, postal code, parents/guardian and number of people in household, marital status) [4]
- Emergency contacts’ phone numbers/emails (no reference, manually added)
- HBM4EU_D7.6_Annex2.1 (selected questions for parents’ education status, country of birth (grandparents, parents and child), language spoken at home, child’s years living in area, employment status, household income) [5]

## Green spaces

- European urban health questionnaire (selected questions for green areas) [6]

## Psychological and mental stress

- **Quality of life in the neighborhood** - European urban health questionnaire (selected questions for quality of life in the neighborhood) [6]
- **Child’s quality of life** - KINDL [7].

## Policies and programs

**Health care access (parents)** - Survey on Income and Living Conditions of Households 2022 (selected questions for health care related goods and services) [4]

Specific external exposome

## Noise

European urban health questionnaire (selected question for noise) [6]

## Chemical contaminants

**Residential environment and home exposures** (e.g. traffic, ventilation, floor materials, heating source, household cleaning activities, cooking methods at home)

- HBM4EU_D7.6_Annex2.1 (selected questions for residential environment and home exposures e.g. traffic, ventilation, floor materials, heating source, household cleaning activities, cooking methods at home) [5]
- HELIX Subcohort (selected questions on Presence of child during cleaning) [8]
- HBM4EU_WP7_2ndPrio (selected questions for cooking methods, garden, pesticides use) [5]

**School exposures (e.g.** recent painting, cleaning practices, pesticide use, ventilation)

- SINPHONIE project
  - Headmaster’s questionnaire – for school characteristics
  - Teacher’s questionnaire – for class characteristics

## Occupation

**Parents’ occupation and occupational exposures** - HBM4EU_D7.6_Annex2.1 (selected questions for parents’ employment and occupational exposures) [5]

## Lifestyle

**Parents’ smoking and alcohol habits**

- HBM4EU_D7.6_Annex2.1 (selected questions for smoking in the house)[5]
- HBM4EU_D7.6_Annex1.1 (selected questions for smoking and alcohol habits) [5]

**Children’s lifestyle**

- HBM4EU_D7.6_Annex2.1 (selected questions for drinking water habits, food storage, physical activity time, time spent at places, cosmetic and hygiene products use, washing hands, activities at home/school) [5]
- Diet
  - IDEFICS 24hDR (2 weekdays & 1 weekend day) [9]
  - HBM4EU_D7.6_Annex2.1 (selected questions for supplements, fast food recent consumption, school lunches) [5]
  - HBM4EU_WP7_2ndPrio (selected questions for fruits/vegetables treatment before consumption, organic food consumption) [5]
- Adherence to Mediterranean diet - KIDMED [10]
- Screen media use - SCREENS questionnaire [11]
- Chronotype assessment - Children's Chronotype Questionnaire [12]
- Sleep - Sleep Disturbance Scale for Children [13]–use only Factors 1, 3, 5
- HELIX Subcohort (selected questions on Exposure to outside air during sleep) [8]
- Exposure to artificial light at night (ALAN) - Indoor ALAN: subjective measure defined as the level of light in the bedroom during sleeping time (reference in the protocol). Response will be a four-digit Likert scale: a) total darkness, b) almost dark, c) dim light, and d) quite illuminated. [14]

Internal exposome

## Basic characteristics

- HBM4EU_D7.6_Annex2.1 (selected questions for child’s age and sex and parental age) [5]
- HBM4EU_D7.6_ Annex1.1 (selected questions for parental sex) [5]

## Intrinsic properties

- HBM4EU_D7.6_Annex1.1 (selected questions for parents’ anthropometrics)[5]
- Question to parents regarding children’s anthropometrics for previous years – advise them to check the child’s medical booklet

## Reproduction-related variables

- IDEFICS Pregnancy and early childhood questionnaire [15]

## Biochemistry and medical indicators

**Children**

- HBM4EU_D7.6_Annex2.1 (selected questions for child’s diseases, medicine use, dental health, artificial material, metallic jewelry, glasses/contact lenses) [5]
- Hearing function – no reference
- Respiratory health - International Study of Asthma and Allergies in Childhood (ISAAC) [16]
- Autism spectrum disorder screening - Autism Spectrum Screening Questionnaire (ASSQ) [17]

**Parents** - HBM4EU_D7.6_Annex1.1 (selected questions for parents’ diseases)[5]

Additional questions

**Questions relevant to urine and saliva sampling** - HBM4EU_D7.3_Annex2.1.3 [5] (selected questions)

Questions relevant for older children (> 9 years old)

**Specific external exposome**

- **Child’s smoking and alcohol habits – relevant for children aged 10-11**
  - HBM4EU_D7.6_Annex2.1 [5] (selected questions for child’s smoking and alcohol habits)

**Internal exposome**

- **Child’s pubertal development – relevant for children aged 9-11**
  - Puberty scale [18]

References

1. Harris PA, Taylor R, Thielke R, Payne J, Gonzalez N, Conde JG. Research electronic data capture (REDCap)—A metadata-driven methodology and workflow process for providing translational research informatics support. J Biomed Inform. 2009;42: 377–381. doi:https://doi.org/10.1016/j.jbi.2008.08.010

2. NHANES. Anthropometry Procedures Manual. 2007. Available: ov/nchs/data/nhanes/nhanes_07_08/manual_an.pdf

3. Haddad N, Andrianou XD, Makris KC. A Scoping Review on the Characteristics of Human Exposome Studies. Curr Pollut Rep. 2019;5: 378–393. doi:10.1007/s40726-019-00130-7

4. Statistical Service of Cyprus. Survey on Income and Living Conditions of Households 2022. Feb 2022. Available: https://www.cystat.gov.cy/en/QuestionnaireList?s=44

5. González-Alzaga B, Hernández AF, Kim Pack L, Iavicoli I, Tolonen H, Santonen T, et al. The questionnaire design process in the European Human Biomonitoring Initiative (HBM4EU). Environ Int. 2022;160: 107071. doi:10.1016/j.envint.2021.107071

6. EURO-URHIS 2. European Urban Health Indicators Part Two: Using indicators to inform policy. 2014 Dec. Available: https://cordis.europa.eu/project/id/223711/reporting

7. Ravens-Sieberer U, Bullinger M. Assessing health-related quality of life in chronically ill children with the German KINDL: first psychometric and content analytical results. Qual Life Res. 1998;7: 399–407. doi:10.1023/A:1008853819715

8. ISGlobal. HELIX Project: Data Inventory (Subcohort). 2014 [cited 20 Jan 2023]. Available: http://www.projecthelix.eu/data-inventory

9. Bammann K, Lissner L, Pigeot I, Ahrens W, editors. Web-Based 24-h Dietary Recall: The SACANA Program. Instruments for Health Surveys in Children and Adolescents. Cham: Springer International Publishing; 2019. doi:10.1007/978-3-319-98857-3

10. López-Gajardo MA, Leo FM, Sánchez-Miguel PA, López-Gajardo D, Soulas C, Tapia-Serrano MA. KIDMED 2.0, An update of the KIDMED questionnaire: Evaluation of the psychometric properties in youth. Front Nutr. 2022;9: 945721. doi:10.3389/fnut.2022.945721

11. Klakk H, Wester CT, Olesen LG, Rasmussen MG, Kristensen PL, Pedersen J, et al. The development of a questionnaire to assess leisure time screen-based media use and its proximal correlates in children (SCREENS-Q). BMC Public Health. 2020;20: 664. doi:10.1186/s12889-020-08810-6

12. Werner H, LeBourgeois MK, Geiger A, Jenni OG. Assessment of Chronotype in Four- to Eleven-Year-Old Children: Reliability and Validity of the Children’s ChronoType Questionnaire (CCTQ). Chronobiol Int. 2009;26: 992–1014. doi:10.1080/07420520903044505

13. Bruni O, Ottaviano S, Guidetti V, Romoli M, Innocenzi M, Cortesi F, et al. The Sleep Disturbance Scale for Children (SDSC) Construct ion and validation of an instrument to evaluate sleep disturbances in childhood and adolescence. J Sleep Res. 1996;5: 251–261. doi:10.1111/j.1365-2869.1996.00251.x

14. Garcia-Saenz A, Sánchez de Miguel A, Espinosa A, Valentin A, Aragonés N, Llorca J, et al. Evaluating the Association between Artificial Light-at-Night Exposure and Breast and Prostate Cancer Risk in Spain (MCC-Spain Study). Environ Health Perspect. 2018;126: 047011. doi:10.1289/EHP1837

15. Bammann K, Lissner L, Pigeot I, Ahrens W, editors. I.Family Pregnancy and Early Childhood Questionnaire. Instruments for Health Surveys in Children and Adolescents. Cham: Springer International Publishing; 2019. doi:10.1007/978-3-319-98857-3

16. Asher, Weiland. The International Study of Asthma and Allergies in Childhood (ISAAC). Clin Exp Allergy. 1998;28: 52–66. doi:10.1046/j.1365-2222.1998.028s5052.x

17. Ehlers S, Gillberg C, Wing L. A screening questionnaire for Asperger syndrome and other high-functioning autism spectrum disorders in school age children. J Autism Dev Disord. 1999;29: 129–141. doi:10.1023/A:1023040610384

18. Carskadon MA, Acebo C. A self-administered rating scale for pubertal development. J Adolesc Health. 1993;14: 190–195. doi:10.1016/1054-139X(93)90004-9
